# SLAP@g-C_3_N_4_ Fluorescent Photocatalytic Composite Powders Enhance the Anti-Bacteria Adhesion Performance and Mechanism of Polydimethylsiloxane Coatings

**DOI:** 10.3390/nano12173005

**Published:** 2022-08-30

**Authors:** Gang Xiong, Zhanping Zhang, Chen Zhang, Yuhong Qi

**Affiliations:** 1 Key Laboratory of Ship-Machinery Maintenance & Manufacture, Dalian Maritime University, Dalian 116000, China; 2 Department of Materials Science and Engineering, Dalian Maritime University, Dalian 116000, China

**Keywords:** fluorescence, graphite phase carbon nitride, photocatalysis, silicone, marine antifouling

## Abstract

Fluorescent antifouling and photocatalytic antifouling technologies have shown potential in the field of marine antifouling. SLAP@g-C_3_N_4_/PDMS (SLAP@CN/PDMS) composite antifouling coatings were designed and prepared using g-C_3_N_4_, sky-blue long afterglow phosphor (SLAP), and polydimethylsiloxane (PDMS). The fluorescence emitted by SLAP under dark conditions was used to excite g-C_3_N_4_ for fluorescent photocatalysis and to prolong the photocatalytic activity of g-C_3_N_4_. Key data were collected by testing and characterization and are presented in this work. The results showed that g-C_3_N_4_ was successfully coated on the SLAP surface and formed a heterogeneous structure. After the composite powder was added to the PDMS coating, the coating maintained low surface energy but enhanced the surface roughness of the coating. The experimental results of degraded Rhodamine B (RhB) showed that SLAP prolonged the g-C_3_N_4_ photocatalytic activity time. The anti-marine bacterial adhesion performance of the coating was investigated by bacterial adhesion experiments. The results showed that SLAP@CN could effectively improve the anti-bacterial adhesion performance of PDMS coating, in which the anti-bacterial adhesion performance of SLAP@CN-2.5/PDMS was improved by nearly 19 times. This antifouling coating introduces fluorescent antifouling, photocatalytic antifouling, and fluorescence-driven photocatalytic antifouling based on the low surface energy antifouling of silicones and achieves “all-weather” fluorescent photocatalytic antifouling.

## 1. Introduction

Fouling organisms (such as bacteria, diatoms, barnacles, mussels, etc.) can cause serious hazards to marine vessels and industrial facilities, leading to serious economic losses and environmental pollution problems [1,2]. Antifouling coatings effectively prevent fouling organisms from adhering to the surface of ships. However, the harmful substances (organotin, cuprous oxide, volatile organic compounds, etc.) released from past antifouling coatings have also caused different degrees of harm to the marine environment [3,4]. Therefore, the development of new environment-friendly antifouling coatings has become a hot topic of research at home and abroad. At present, the research on environmentally friendly antifouling coatings is divided into two main categories: developing non-toxic antifouling agents and fouling-release antifouling coatings with non-single antifouling mechanisms [5]. However, the release rate and amount of non-toxic antifouling agents are difficult to control, and the preparation is more costly and difficult. Therefore, it becomes more economical and easier to develop fouling-release antifouling coatings with multiple antifouling mechanisms. Among the fouling-release antifouling coatings, silicone antifouling coatings have been widely studied because of their good antifouling properties and non-pollution to the environment [6]. Polydimethylsiloxane (PDMS) has low surface energy and low modulus of elasticity, which can effectively inhibit fouling organisms’ adhesion, weaken fouling organisms’ adhesion strength on the surface of the coating, and detach the organisms attached to the surface of the ship by the scouring of seawater. In contrast, it is difficult for fouling organisms to detach under static conditions and finally adhere to the ships’ surface [7]. The static antifouling performance of silicone antifouling coatings has been improved by introducing semiconductor photocatalytic materials, polymers, micro-nanoparticles, and silicone oils into silicones [8,9,10,11].

In recent years, photocatalytic materials have shown great potential for environmental protection and energy utilization because of their environmental friendliness and high efficiency [12,13]. Meanwhile, photocatalytic materials have been proved to have superior anti-bacterial properties [14,15,16]. When photocatalytic materials are irradiated by incident light with energy greater than their forbidden bandwidth, the valence band electrons are excited to the conduction band, generating electron–hole pairs and undergoing redox reactions. In addition, the reactive oxygen species (ROS) generated in this process can effectively decompose organic matter and kill microorganisms [17]. When ships are immersed in seawater, the first two stages of fouling organism attachment are mainly the attachment of organic molecules (proteins, polysaccharides, etc.) and bacterial microorganisms [3,18]. Therefore, the ROS produced by the photocatalytic reaction can effectively prevent organic molecules and bacterial microorganisms from adhering to the coating surface, enhancing the antifouling performance of the coating. Among the many photocatalytic materials, TiO_2_, ZnO, g-C_3_N_4_, CdS, and WO_3_ are widely used in the field of photocatalysis [19]. TiO_2_ is commonly used for the photocatalytic degradation of organic pollutants and decomposition of water because of its high chemical stability and activity [20]. However, the wide band gap of TiO_2_ leads to its low photocatalytic activity under the irradiation of visible light and also limits its application [21,22]. Therefore, g-C_3_N_4_, which has photocatalytic activity under visible light at wavelengths less than 460 nm, shows promising applications. Previous studies have shown that g-C_3_N_4_-based photocatalytic materials show good sterilization and toxicity-killing activity [23]. Meanwhile, g-C_3_N_4_ is also a photocatalytic material with a narrow forbidden band (~2.7 eV), chemical stability, easy availability, higher photocatalytic efficiency, and no pollution to the environment [24,25]. However, pristine g-C_3_N_4_ presents an aggregated stacked state, and its low specific surface area and high complexation rate of photogenerated electrons and holes limit its application in photocatalysis [26]. Usually, the modification of g-C_3_N_4_ by ion doping, liquid phase exfoliation, construction of heterostructures, and morphology modulation is a common method to improve its photocatalytic activity [27]. It is worth noting that the photocatalytic reactions occurring in photocatalytic materials can only take place during the daytime due to the diurnal shift, resulting in a low utilization rate of photocatalytic materials. Therefore, providing g-C_3_N_4_ with a longer time light source is an effective way to enhance its photocatalytic performance.

Long afterglow phosphor is a functional material that can store light and emit light in a long-term cycle, and it can provide a suitable light source for g-C_3_N_4_ in a dark environment. Among them, aluminate and silicate systems with long afterglow phosphors are more widely used. Aluminate long afterglow phosphors have good stability of UV radiation resistance and long afterglow time, but their water resistance is poor. Silicate long afterglow phosphors are chemically stable and also have good afterglow properties, UV radiation resistance, and water resistance [28,29]. Compared with aluminate long afterglow phosphors, silicate long afterglow phosphors are more chemically stable and have better water resistance. Therefore, silicates are more suitable for applications in marine environments. It should also be noted that the ability of g-C_3_N_4_ to utilize fluorescence for photocatalytic reactions presupposes that the luminescence wavelength of the long afterglow phosphor and the light absorption wavelength of g-C_3_N_4_ need to match. The range of emission wavelengths of Sr_2_MgSi_2_O_7_:(Eu^2+^, Dy^3+^) is approximately from 420–550 nm, a range of wavelengths that can directly excite g-C_3_N_4_ [30]. Currently, there is already g-C_3_N_4_ compounded with aluminate (SrAl_2_O_4_:(Eu^2+^, Dy^3+^), Sr_4_Al_14_O_25_:(Eu^2+^, Dy^3+^)), and silicate (Sr_2_MgSi_2_O_7_:(Eu^2+^, Dy^3+^)) long afterglow phosphors have been studied [28,31,32]. These studies confirmed that fluorescence can drive photocatalytic reactions. However, these studies have only been applied to the degradation of organic matter and pollutants. To date, no study has emerged on the application of g-C_3_N_4_ and long afterglow phosphors compounded for marine antifouling. Not only that, but past studies have shown that long afterglow phosphors have antifouling effects and that both the luminescence intensity and luminescence color of long afterglow phosphors have an effect on the anti-bacterial properties of the coatings [33]. Among them, sky-blue high-brightness long afterglow phosphor (Sr_2_MgSi_2_O_7_:(Eu^2+^, Dy^3+^) has the best anti-bacterial properties. Therefore, it is a valuable and promising work to compound g-C_3_N_4_ with Sr_2_MgSi_2_O_7_:(Eu^2+^, Dy^3+^) and apply it in the field of marine antifouling.

In this study, SLAP@g-C_3_N_4_ (SLAP@CN) composite powder with the fluorescence-driven photocatalytic function was designed and prepared. Then, a new SLAP@g-C_3_N_4_/PDMS (SLAP@CN/PDMS) composite antifouling coating was prepared by adding it to silicone antifouling coating. The successful preparation of SLAP@CN was confirmed by the characterization of the composite powder by XRD, FT-IR, SEM, and TEM. The optical properties of the composite powders and coatings were tested by UV–Vis, PL, and weak fluorescence photometry. The photocatalytic properties of the composite powder under light and dark conditions were evaluated by degradation of Rhodamine B (RhB). The resistance of the coating to the adhesion of marine bacteria was evaluated by a marine bacterial adhesion test. The experimental results show that the composite coating has good resistance to organic pollutants and bacterial adhesion. The design of this new composite coating with multiple antifouling mechanisms provides a new strategy for the development of environmentally friendly marine antifouling coatings.

## 2. Materials and Methods

### 2.1. Materials

Because the fluorescence emission wavelength of long afterglow phosphor needed to match the absorption wavelength of g-C_3_N_4_ and has good water resistance, we chose sky-blue long afterglow phosphor (Sr_2_MgSi_2_O_7_:(Eu^2+^, Dy^3+^), SLAP) produced by Dalian Luminous Technology Co. Since the specific surface area of g-C_3_N_4_ prepared from urea was larger, urea (AR, 98%) produced by Fuchen Chemical Reagent Co., Ltd. (Tianjin, China) was purchased as a precursor for the preparation of g-C_3_N_4_. Ten thousand molecular weight polydimethylsiloxane (PDMS) was selected as the film-forming substance for the preparation of the coating and purchased from Shandong Dayi Chemical Co. (Shandong, China). Isopropanol (AR, ≥99.7%), anhydrous ethanol (AR, ≥99.7%), and ethyl orthosilicate (TEOS, AR, 98%) were obtained from Tianjin Damao Chemical Reagent Factory (Tianjin, China). Pyrogenic silica (AS-150) was obtained from Shenyang Chemical Co. Anti-settling agent DeuRheo202P was obtained from Hemmings Deqian Chemical Co. (Changzhou, China). The organic solvents xylene (AR, 99%) and methyl isobutyl ketone (MIBK, AR, 99%) were purchased from Tianjin Comio Chemical Reagent Co. The catalyst bismuth neodecanoate (DY-20) was purchased from Shanghai Deyin Chemical Co. (Shanghai, China), and the bismuth content was 20 ± 0.5%.

### 2.2. Preparation of SLAP@CN/PDMS Composite Antifouling Coatings

#### 2.2.1. Preparation of Flake g-C_3_N_4_

**Figure 1:**
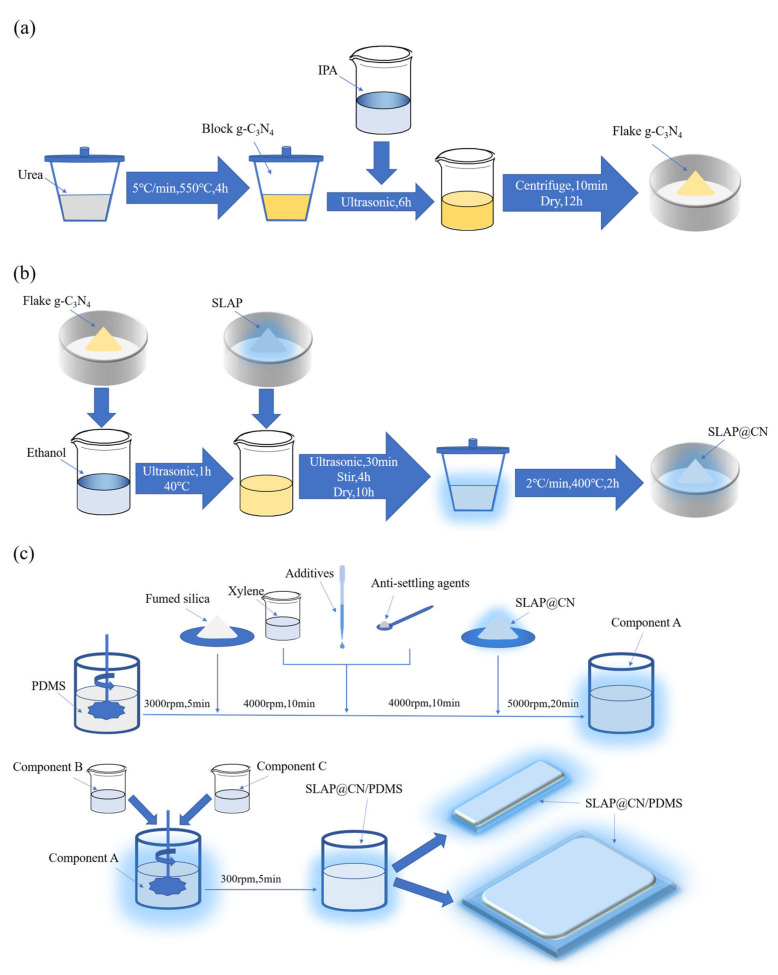
(**a**) Preparation flow chart of flake g-C_3_N_4_; (**b**) preparation flow chart of SLAP@CN; (**c**) preparation flow chart of SLAP@CN/PDMS composite antifouling coatings.

The flake g-C_3_N_4_ was prepared by the thermal polymerization and liquid phase exfoliation methods, and the preparation process is shown in Figure 1a. First, urea (15 g) was taken into a crucible (50 mL) with a lid, and the crucible was placed in a box-type high-temperature heating furnace (KSL1700X, Hefei Kejing Materials Technology Co., Ltd., Hefei, China) and heated to 550 °C at 5 °C/min. After holding for 4 h and cooling naturally to room temperature, a yellowish block of g-C_3_N_4_ (about 0.4 g) was obtained. Then, g-C_3_N_4_ (0.1 g) was added into IPA (200 mL) and sonicated for 6 h at a rated power of 800 W using a cell crusher (JY92-IID, Ningbo Haishu Wufang Ultrasonic Equipment Co., Ltd., Ningbo, China) and then centrifuged for 10 min at 3000 rpm using a centrifuge (TDL-50C, Shanghai Anting Scientific Instruments Co., Ltd., Shanghai, China) after sonication. The centrifuged g-C_3_N_4_ was dried in a vacuum drying oven (DZ-1BCIV, Tianjin Teste Instruments Co., Ltd., Tianjin, China) at a temperature of 60 °C for 12 h to obtain g-C_3_N_4_ in flakes.

#### 2.2.2. Preparation of SLAP@CN

Four SLAP@CN fluorescent photocatalytic composite powders with g-C_3_N_4_ masses of x% of SLAP (x = 1, 2.5, 4, 6), respectively, were designed and prepared. The series of SLAP@CN composite powders was prepared after three stages of solvent ultrasonic dispersion, mechanical stirring and mixing, and high-temperature calcination, and the preparation process is shown in Figure 1b. Firstly, 0.25 g/0.625 g/1 g/1.5 g of g-C_3_N_4_ was added into the beaker with 100 mL of ethanol and ultrasonically dispersed at 40 °C for 1 h. Then, 25 g of SLAP was added separately and continued to be ultrasonically dispersed for 30 min. Next, the dried powder was put into a crucible with a lid and placed in a box-type high-temperature heating furnace, heated up to 400 °C at 2 °C/min, and calcined for 2 h. Finally, the mixture cooled to room temperature to obtain SLAP@CN powder.

#### 2.2.3. Preparation of SLAP@CN/PDMS, SLAP/PDMS, and g-C_3_N_4_/PDMS Composite Antifouling Coatings

SLAP@CN/PDMS, SLAP/PDMS, and g-C_3_N_4_/PDMS composite antifouling coatings were designed and prepared. The composite antifouling coatings were composed of three components, namely components A, B, and C. The composite antifouling coating was prepared by a three-step method, and the preparation process is shown in Figure 1c. In the first step, PDMS (100 g) was first added to the stirring vessel. The vessel was fixed in the sanding and dispersion stirring multi-purpose machine (BGD750, Guangzhou BGDA Experimental Instruments Co., Ltd., Guangzhou, China) at 3000 rpm for 5 min. Then, fumed silica (10 g) was added to the vessel and stirred at 4000 rpm for 10 min. Then, solvent, auxiliary, and anti-sinker were added to the container and stirred at 4000 rpm for 10 min. Finally, SLAP@CN (SLAP or g-C_3_N_4_) with a mass of 20 g was added to the container and stirred at 5000 rpm for 20 min to obtain component A. In the second step, add component B to component A and stir well at 300 rpm, and then quickly add component C and stir at 300 rpm for 5 min to obtain the composite antifouling coating. In the third step, the resulting composite antifouling coating was quickly applied and poured onto a slide and a PTFE mold and cured at room temperature for 8 h to obtain SLAP@CN/PDMS. SLAP/PDMS and g-C_3_N_4_/PDMS composite antifouling coatings were prepared, where the masses of SLAP and g-C_3_N_4_ added were 20 g and 0.8 g, respectively. Meanwhile, PDMS coatings without the composite powder were prepared as a control group.

### 2.3. Characterization

#### 2.3.1. Chemical and Crystal Structure Analysis

The physical phase composition of g-C_3_N_4_, SLAP, and SLAP@CN was analyzed using an X-ray diffractometer (XRD, Bruker D8 Advance). The test range was 10–90° with a step size of 0.02° and a scan speed of 8°/min with a copper target.

The chemical structures of g-C_3_N_4_, SLAP, and SLAP@CN were analyzed using Fourier transform infrared spectroscopy (FTIR, Nicolet IS5). The dried powders were tested with KBr compressions in the wave number range of 400–4000 cm^−1^ with a resolution of 4.0 cm^−1^ and 32 scans.

#### 2.3.2. Analysis of Microstructure

The samples were dispersed in anhydrous ethanol, dried, and fixed on the conductive gel for testing. The microstructure, elemental content, and distribution of g-C_3_N_4_, SLAP, and SLAP@CN were observed using a field emission scanning electron microscope (SEM, HITACHI SU5000) and an energy-dispersive X-ray spectrometer (EDS, Oxford instruments Ultim Max). The acceleration voltage selected for the SEM was 20.0 kV.

The microstructure and lattice stripes of g-C_3_N_4_ and SLAP@CN were observed using field emission transmission electron microscopy (TEM, Tecnai G2 F20). The samples were ultrasonically dispersed in anhydrous ethanol for 5 min and placed on a copper grid to dry. The tests were performed at an operating voltage of 200 kV.

#### 2.3.3. Spectral and Illuminance Analysis

The absorbance of g-C_3_N_4_, SLAP, and SLAP@CN was measured using a UV–Vis spectrophotometer (UV–Vis, UV 3600 Plus), and the band gap was calculated. The test wavelength range was 200–800 nm.

The emission spectra of g-C_3_N_4_, SLAP, and SLAP@CN were tested using a steady-state fluorescence spectrometer (PL, F4600). The excitation wavelength was 370 nm, and the test wavelength range was 400–650 nm.

The afterglow decay curves of the coatings were tested using a weak fluorescence photometer (ST-900). The test light source was a fluorescent lamp with a light intensity of 2150 lx at a distance of 20 cm from the surface of the specimen. After 20 min of illumination, the light source was turned off and the afterglow illumination of the specimen was measured at different intervals for 120 min.

#### 2.3.4. Surface Roughness

The microscopic morphology of the coating surface was observed using an OLS4000 laser confocal microscope (CLSM), and the surface roughness of the coating was determined using LEXT software (LEXT 2.1.3, OLYMPUS, Japan). The surface roughness of three specimens was tested and the average value was taken as the surface roughness of the coating.

#### 2.3.5. Wettability and Surface Energy

The water contact angle (WCA) of the coating was measured using a contact angle measuring instrument (JC2000C), and the contact angle values were measured using contact angle measuring software (JC2000C-CG400). The Owens two-liquid method was also used to calculate the surface energy (SE) of the coating [34]. Test reagents were selected from deionized water and diiodomethane (CH_2_I_2_), and 3 μL of reagent was dropped on the upper surface of the specimen at a time. The contact angle of each specimen was tested at five points, and the average value was taken as the contact angle value of the specimen.

### 2.4. Photocatalytic Performance Test

Rhodamine B (RhB) in industrial dye wastewater presents high concentration and is not easily degradable. In addition, it is difficult to completely degrade RhB by conventional treatment techniques. Therefore, RhB was chosen as the target pollutant for the photocatalytic activity test of the composite powder. The experimental light source was a 300 W xenon lamp equipped with a 420 nm filter, and the incident light intensity was about 150 mW/cm^2^. For each experiment, 200 mg of the composite powder was added to 10 mg/L of RhB (100 mL) solution for the reaction. The control group used 5 mg g-C_3_N_4_, and the addition amount was the same as that of g-C_3_N_4_ in SLAP@CN-2.5. To evaluate the photocatalytic activity of the composite powders under dark conditions, the experiments were divided into light stage and dark stage. Before turning on the light source, the suspension was stirred magnetically for 30 min in the dark to reach adsorption–desorption equilibrium. The light source was then turned on and the suspension was stirred under xenon light for 40 min. After 40 min, the light source was turned off and the suspension was transferred to a dark environment and stirred for 80 min. Every 20 min, 4 mL of the suspension was centrifuged, and then the concentration of RhB was measured using UV–Vis.

### 2.5. Anti-Marine Bacteria Adhesion Performance Test

To simulate the anti-marine bacteria adhesion performance of the coating in the actual marine environment, fresh seawater rich in marine bacteria was selected as the bacterial solution for marine bacterial adhesion experiments. In the first step, the specimens were immersed in fresh seawater for 24 h at 28 °C (12 h dark and 12 h light). In the second step, the specimens were rinsed in 50 mL sterilized seawater at the end of immersion, and then the bacteria on the surface of the specimens were brushed into the sterilized seawater and diluted 10^−6^ times. In the third step, 10 μL of the diluted bacterial solution was taken and spread evenly on the solid medium. Finally, the medium was placed in a biochemical incubator at a temperature of 25 °C for 48 h. After 48 h, the colonies growing on the medium were photographed and recorded, and the bacterial adherence rate was calculated using Image-Pro Plus software (Image-Pro Plus 6.0, MEDIA CYBERNETICS, Rockville, MD, USA).

## 3. Results and Discussion

### 3.1. Crystal Phase Composition and Chemical Structure Characteristics

**Figure 2:**
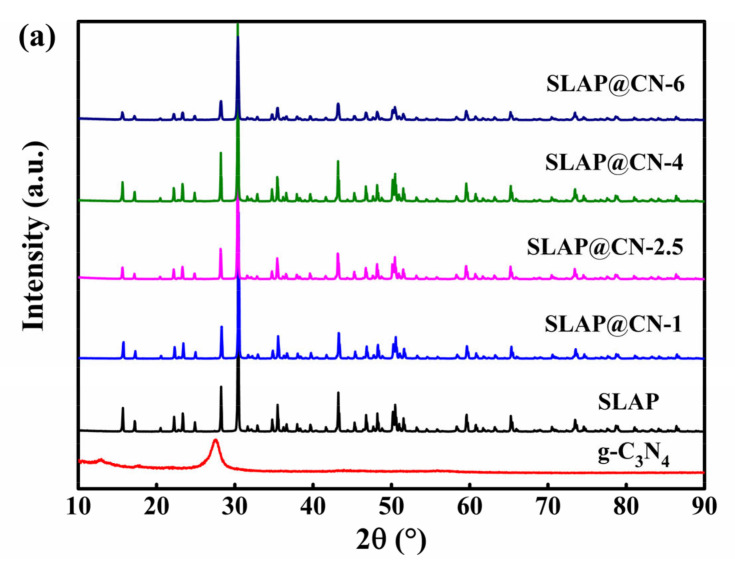

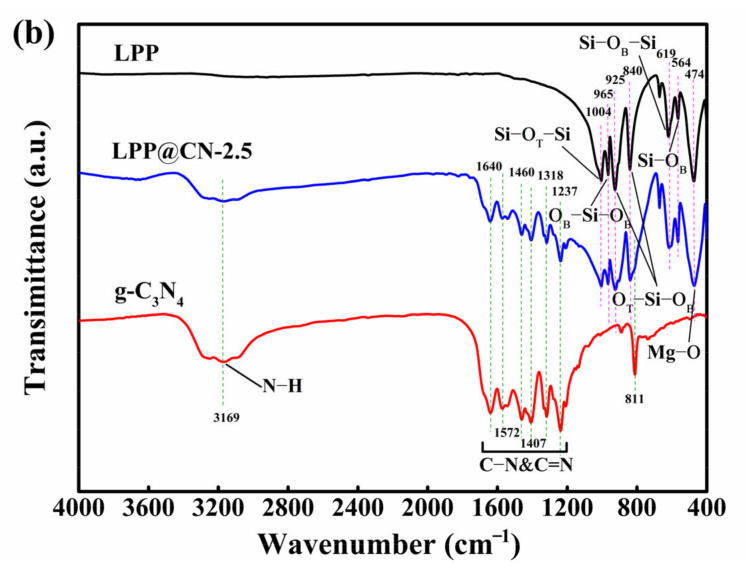
(**a**) XRD patterns of g-C_3_N_4_, SLAP, and SLAP@CN composite powders; (**b**) FTIR spectra of g-C_3_N_4_, SLAP, and SLAP@CN-2.5.

The measured XRD patterns of g-C_3_N_4_, SLAP, and SLAP@CN are shown in Figure 2a. Two distinct characteristic peaks appeared in g-C_3_N_4_ at 12.9° and 27.4°, corresponding to the (100) and (002) crystal planes, respectively [35]. The weaker diffraction peak at 12.9° corresponds to the diffraction of three in-plane heterocycles linked by triple nitrogen, and the stronger diffraction peak at 27.4° corresponds to the diffraction formed by the inter-plane stacking [36]. The disappearance of the characteristic peak of g-C_3_N_4_ in the spectrum of SLAP@CN is mainly caused by the following three factors. First, the content of g-C_3_N_4_ (<6%) is too small compared with SLAP. According to previously reported composites of long afterglow phosphors with g-C_3_N_4_, the composite powder still did not show the characteristic peak of g-C_3_N_4_ when the content of g-C_3_N_4_ was 20% [32]. Secondly, g-C_3_N_4_ was exfoliated into smaller size flakes by ultrasonication as well as secondary calcination of g-C_3_N_4_. Meanwhile, the significantly higher crystallinity of SLAP than g-C_3_N_4_ also contributes to this result. For SLAP@CN, the diffraction peaks are the same, indicating that the composite powders have the same crystal structure, and the high temperature did not lead to the transformation of their crystal structures.

The Fourier transform infrared (FTIR) spectra of g-C_3_N_4_, SLAP, and SLAP@CN-2.5 are shown in Figure 2b. The analysis shows that only characteristic peaks of g-C_3_N_4_ and SLAP exist in the infrared spectrum of SLAP@CN, and no new peaks appear. It shows that the chemical structure of g-C_3_N_4_ and SLAP does not change after compounding, and they are successfully compounded through surface–interface contact. In the infrared spectrum of g-C_3_N_4_, the special plane bending vibration containing the triazine unit corresponds to the absorption peak at 811 cm^−1^. The absorption peak from 1200–1700 cm^−1^ is generated by the typical stretching vibration of C-N, C=N on carbon–nitrogen aromatic heterocycles. The broad absorption peak at 3000–3500 cm^−1^ is the stretching vibration of the amino group and water [37,38]. In the infrared spectrum of SLAP, the absorption peak at 1004 cm^−1^ corresponds to the asymmetric telescopic vibration of the Si-O_T_-Si group, and the absorption peak of 619cm^−1^ corresponds to the symmetrical telescopic vibration of the Si-O_B_-Si group. Among them, there are two types of O atoms, which are divided into O_T_ (bridge oxygen) and O_B_ (non-bridge oxygen). The absorption peak at 965 cm^−1^ is attributed to the symmetric stretching motion of the O_B_-Si-O_B_ group, and the absorption peaks at 925 cm^−1^ and 840 cm^−1^ correspond to the asymmetric stretching motion of the O_T_-Si-O_B_ group. The absorption peak at 564 cm^−1^ corresponds to the bending vibration of Si-O_B_, and the absorption peak at 474 cm^−1^ is generated by the stretching vibration of the Mg-O bond [28,29].

### 3.2. Morphology

**Figure 3:**
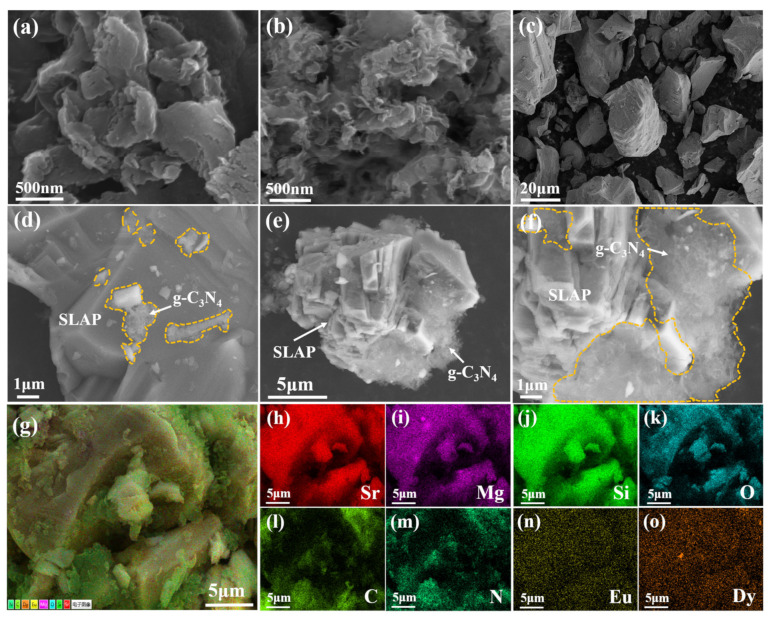
(**a**) SEM image of pristine g-C_3_N_4_; (**b**) SEM image of g-C_3_N_4_ after liquid phase exfoliation; (**c**) SEM image of SLAP; (**d**) SEM image of SLAP@CN-2.5; (**e**–**o**) SEM images of SLAP@CN-6 and their corresponding EDS element distributions.

**Figure 4:**
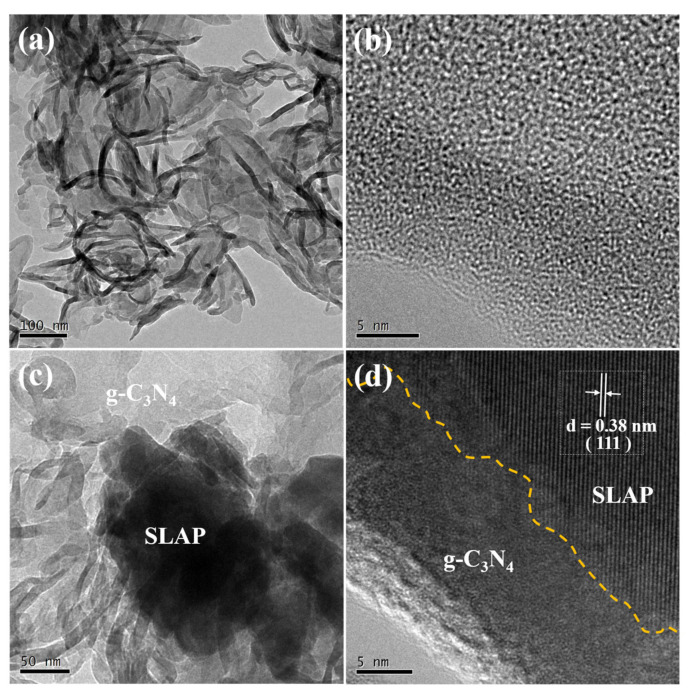
(**a**) TEM image of g-C_3_N_4_; (**b**) high-resolution TEM image of g-C_3_N_4_; (**c**) TEM image of SLAP@CN-6; (**d**) high-resolution TEM image of SLAP@CN-6.

To verify the morphology and structure of SLAP@CN, the composite powders were observed and analyzed by scanning electron microscopy (SEM) and transmission electron microscopy (TEM). The SEM test results are shown in Figure 3. Figure 3a,b show the microstructures of g-C_3_N_4_ before and after liquid phase exfoliation, respectively. The pristine g-C_3_N_4_ exhibits a block structure, while the exfoliated g-C_3_N_4_ exhibits a curled sheet-like structure. This result confirms that g-C_3_N_4_ achieves exfoliation in IPA. The exfoliated g-C_3_N_4_ is flake-like, and the specific surface area is increased. The larger specific surface area increases the photocatalytic active sites, which is beneficial to improving the photocatalytic reaction efficiency [39]. Figure 3c shows the surface topography of SLAP, which is irregular in shape. Figure 3d–f are the surface morphologies of SLAP@CN-2.5 and SLAP@CN-6, respectively. g-C_3_N_4_ is randomly coated on the SLAP surface, and the more content of g-C_3_N_4_ in the composite powder there is, the larger the area covered by the SLAP surface is. The elemental composition of the SLAP@CN-6 composite powder was further determined by energy-dispersive X-ray spectroscopy (EDS). The EDS map in Figure 3g is shown in Figure 3h–o. The results show that C and N elements are distributed on the surface of SLAP, which proves that g-C_3_N_4_ is successfully coated on the surface of SLAP. The microscopic morphology and lattice fringes were further observed by TEM, as shown in Figure 4. In Figure 4a, g-C_3_N_4_ is in a transparent and black state, and g-C_3_N_4_ becomes flake-like after peeling, so it is in a transparent state. Figure 4b shows the high-resolution microstructure of g-C_3_N_4_. Figure 4c is the TEM image of SLAP@CN-6, the black opaque part is SLAP, and the transparent part is the surface-coated g-C_3_N_4_. Figure 4d is the high-resolution TEM image of SLAP@CN-6. The lattice fringes of SLAP can be observed. The lattice fringes of SLAP have a spacing of 0.38 nm, corresponding to the (111) crystal plane. It can also be seen that there is an obvious crossover heterogeneous interface between g-C_3_N_4_ and SLAP, which also indicates that g-C_3_N_4_ is tightly attached to the surface of SLAP, confirming that SLAP forms a SLAP@CN complex structure with g-C_3_N_4_.

### 3.3. Optical Performance

**Figure 5:**
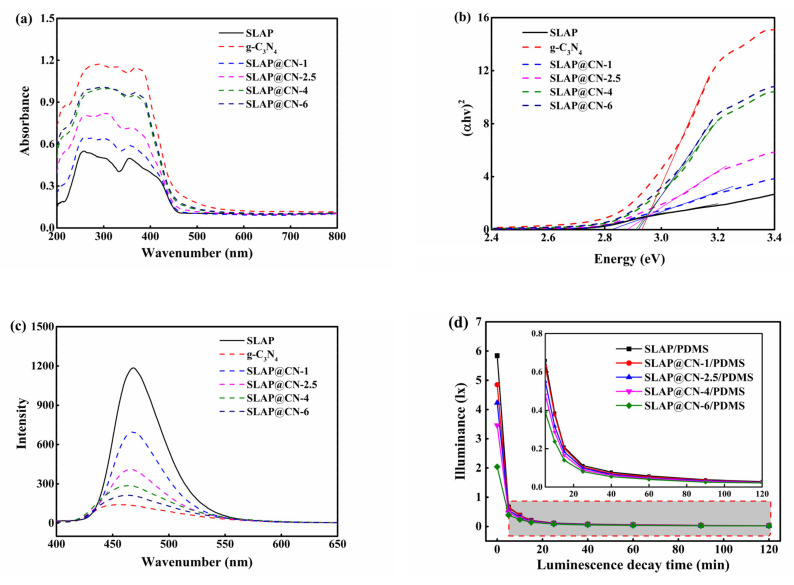
(**a**) UV–Vis spectra of g-C_3_N_4_, SLAP, and SLAP@CN composite powders; (**b**) E_g_ of g-C_3_N_4_, SLAP, and SLAP@CN composite powders; (**c**) PL of g-C_3_N_4_, SLAP, and SLAP@CN composite powders; (**d**) afterglow decay curves of SLAP/PDMS and SLAP@CN/PDMS composite coatings.

The light absorption properties of SLAP, g-C_3_N_4_, and SLAP@CN were analyzed using UV–Vis, as shown in Figure 5a. The results show that SLAP has a weaker absorption capacity for UV and visible light than g-C_3_N_4_ and SLAP@CN. The absorption edge of g-C_3_N_4_ is located near 461 nm and that of SLAP is located near 472 nm. In addition, that of the composite powder is located between 463 and 470 nm. Because of the interaction between SLAP and g-C_3_N_4_, the light absorption intensity increased with the increase of g-C_3_N_4_ content in the composite powder. It indicates that g-C_3_N_4_ can enhance the absorption of visible light by SLAP. SLAP then has better absorption of visible light, which is beneficial to enhancing its luminescence efficiency. Usually, the light absorption ability of photocatalytic composite powders may be related to their energy band structure [40]. The band gap (E_g_) of the powder was calculated according to Tauc plots *(αhv)^2^* = *A(hv* − E_g_*)* [41], which obtained the results as shown in Figure 5b, where *α*, *A*, *h*, *v*, and E_g_ are the absorption coefficient, constant, Planck’s constant, optical frequency, and band gap, respectively. The results show that the E_g_ of SLAP and g-C_3_N_4_ are about 2.74 eV and 2.92 eV, and the results are similar to those previously reported in the literature [42,43]. The E_g_ of composite powder is in the range of 2.82 eV to 2.90 eV. It can be seen that the E_g_ of the SLAP@CN composite powder increases with the increase of g-C_3_N_4_ content in SLAP@CN, which is consistent with its light absorption results. The E_g_ of g-C_3_N_4_ after liquid phase exfoliation increased compared to the original g-C_3_N_4_ (~2.7 eV) [44]. The increased E_g_ is due to the quantum localization effect caused by the increased specific surface area of g-C_3_N_4_ [45]. With E_g_, the empirical formula proposed by Butler and Ginley can be used [46]. We calculated the conduction band and valence band energy levels as −1.23 eV and 1.69 eV, respectively.

The emission spectra (PL) of the materials were tested under an excitation light source at 370 nm, as shown in Figure 5c. g-C_3_N_4_ and SLAP emission peaks were located at 460 nm and 468 nm, respectively, and the emission peaks of the SLAP@ CN composite powder were located from 463–467 nm. The wavelength of the emission peak becomes shorter with the increase of g-C_3_N_4_ content in the composites, and the luminescence intensity decreases with the increase of g-C_3_N_4_ content. The decrease in the intensity of the emission peak is because g-C_3_N_4_ coated on the surface of SLAP decreases the transmittance of the light source, resulting in a decrease in the light absorption of the covered part. Similarly, the light source emitted by SLAP will be reduced or blocked. Therefore, the luminescence intensity of the composite powder will be reduced to different degrees. In addition, the photoluminescence spectrum originates from the compounding of photogenerated electron–hole pairs. Usually, the higher the intensity of the emission peak is, the higher the chance of photogenerated carrier complexation increases [47]. The decrease in emission peak intensity of the composite powder may also be caused by the suppression of photogenerated carrier complexation after the powder is compounded, and the inhibition of photogenerated carrier complexation can effectively improve photocatalytic activity. In addition, the absorption wavelength of g-C_3_N_4_ and the emission wavelength of SLAP show that the wavelength range of SLAP-emitted light is from 420–530 nm, and g-C_3_N_4_ can absorb the light emitted by SLAP for a photocatalytic reaction. It is further demonstrated that the SLAP@ CN composite powder can rely on SLAP for a fluorescent photocatalytic reaction.

The afterglow time and the afterglow intensity of the composite powder are the key factors to continue the photocatalysis. The afterglow decay curve of the test coating after 20 min of irradiation at 2150 lx light intensity is shown in Figure 5d. The afterglow intensity decreases sharply in the first 5 min, and it can be seen by the enlarged graph that the afterglow decay rate after 5 min gradually becomes slower. The luminous intensity of SLAP/PDMS is the largest, with an initial luminous intensity of 5.84 lx. Compounding SLAP with g-C_3_N_4_ will affect the luminous intensity of SLAP, and the luminous intensity of the coating decreases with the increase of the added g-C_3_N_4_ content. It is noteworthy that the ratio of g-C_3_N_4_ and SLAP in the composite powder has a great influence on light absorption and emission. This also directly affects the luminescence performance and photocatalytic performance of the composite powder under light and dark conditions. Therefore, it is necessary to pay attention to the ratio of g-C_3_N_4_ and SLAP in the composite powder.

### 3.4. Surface Performance of Antifouling Coatings

**Figure 6:**
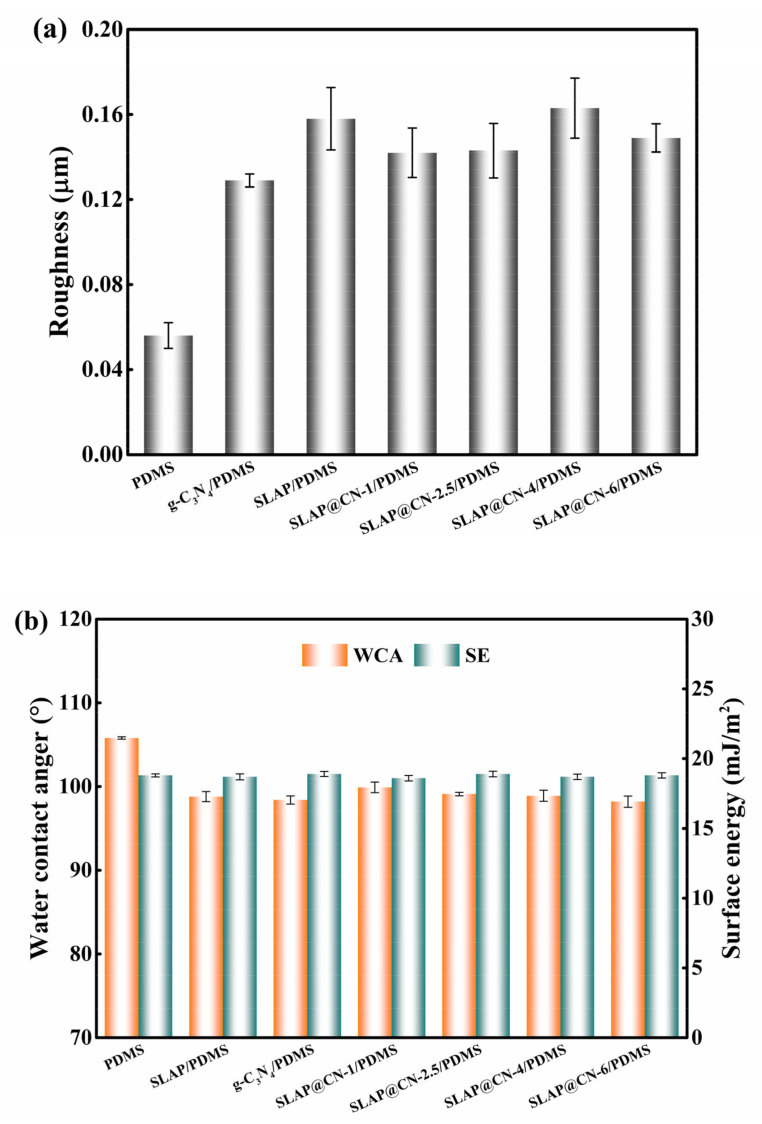
(**a**) Surface roughness of the coating; (**b**) water contact angle and surface energy of the coating.

The surface properties of the coatings were characterized by testing the surface roughness, water contact angle (WCA), and surface energy (SE) of the coatings, as shown in Figure 6. The roughness results in Figure 6a show that the addition of SLAP, g-C_3_N_4_, and SLAP@CN powders increased the surface roughness of the coating. This is due to the microscopic bumps and depressions on the surface of the coating caused by the addition of the composite powders. The WCA of PDMS is 105.8 ± 0.14°, as seen in Figure 6b, and the WCA of the coating decreases by 5.9–7.6° with the addition of the composite powders. According to the Cassie model theory [48], an increase in coating roughness leads to an increase in the WCA. However, the results show that the WCA of the composite coating decreased. The decrease in WCA is mainly because the incorporation of SLAP, g-C_3_N_4_, and SLAP@CN into the coating introduces hydroxyl and amino groups, and this polar group leads to an increase in the hydrophilicity of the coating. Although the WCA of the coating decreased, the coating remains hydrophobic. This is due to the fact that after adding the composite powder to PDMS, the composite powder is uniformly dispersed throughout PDMS, and the contact surface of the composite coating with water is enriched with PDMS. Therefore, the wettability of the composite coating is mainly influenced by the PDMS coating. In addition, the addition of the powder did not lead to an increase in the surface energy of the composite coating. The SE of the coating was at a low value with a magnitude between 18.6 mJ/m^2^ and 18.9 mJ/m^2^, and the SE was lower than 25 mJ/m^2^ for the pure silicone coating. It is known from previous reports that the low SE of the coating is beneficial to enhance the fouling detachment performance of the coating [49].

### 3.5. Photocatalytic Performance

**Figure 7:**
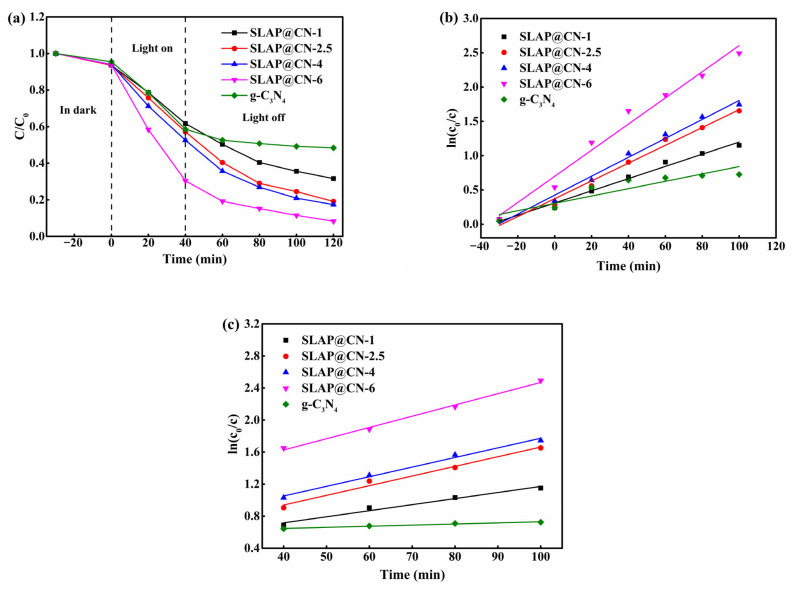
(**a**) Concentration change curves of RhB degradation by g-C_3_N_4_ and SLAP@CN under visible light irradiation; (**b**) degradation efficiency of RhB at full stage; (**c**) degradation efficiency of RhB after turning off the light source.

The photocatalytic activity of g-C_3_N_4_ and SLAP@CN was evaluated by photocatalytic degradation experiments of RhB under visible light, as shown in Figure 7. It was shown that the photocatalytic degradation efficiency increased with the increase of g-C_3_N_4_ content in the SLAP@CN composite powder when the light source was turned on. Since the content of g-C_3_N_4_ in SLAP@CN-2.5 was equal to that of g-C_3_N_4_ in the comparison sample, the photocatalytic efficiency was close. After turning off the light source, the composite powder exhibited a higher sustained degradation of RhB, while RhB mainly underwent self-degradation in the control sample containing only g-C_3_N_4_. Therefore, it is indicated that SLAP in the composite powder provides a blue light source for g-C_3_N_4_ and is responsible for the maintenance of the photocatalytic activity of the composite powder. In addition, the degradation of RhB by the composite powder satisfies the primary kinetic theory *ln(C_0_/C)* = *kt*, where C_0_ is the initial concentration of RhB solution after reaching adsorption equilibrium, C is the concentration of RhB solution at time t, and k is the degradation reaction rate constant. Figure 7b shows the degradation rate of the whole stage, and the degradation rate of SLAP@CN is greater than that of g-C_3_N_4_, and the degradation rate is increased by up to 3.5 times. Figure 7c shows the degradation rate after turning off the light source, and the degradation rate is increased by 10.1 times. Therefore, SLAP can effectively prolong the time of g-C_3_N_4_ photocatalytic activity and enhance photocatalytic efficiency. Similar literature has reported that this composite powder has good photocatalytic stability, and the photocatalytic performance did not decrease significantly through five RhB degradation experiments [50].

### 3.6. Anti-Marine Bacteria Adhesion Properties of Coatings

**Figure 8:**
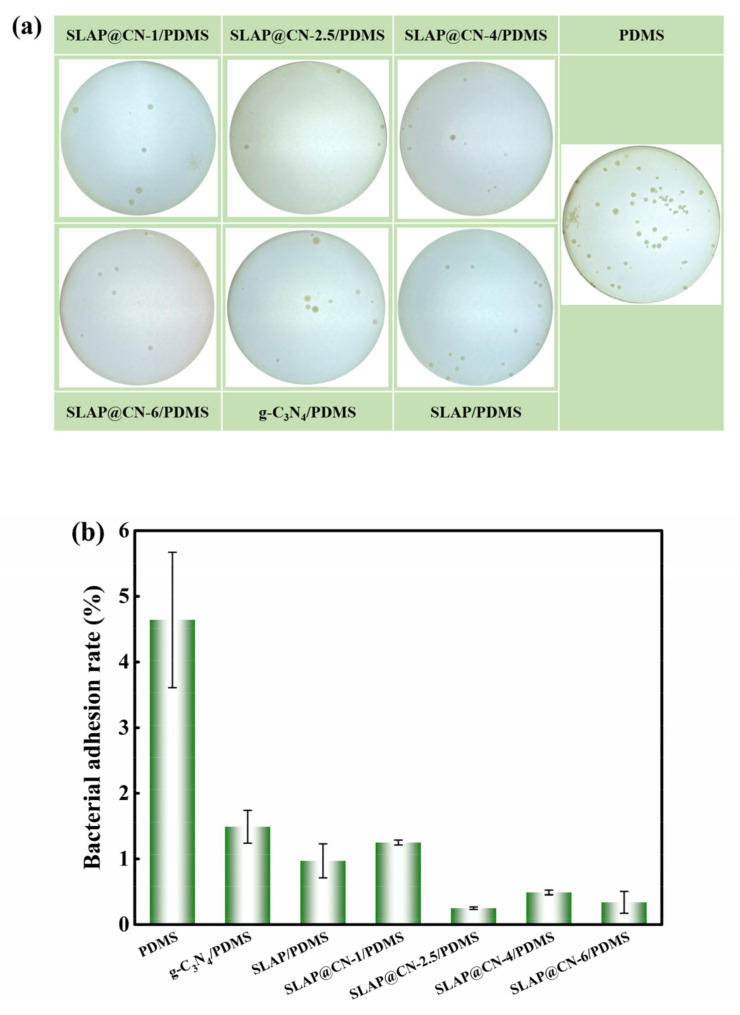
(**a**) Photograph of colony attachment of the coating; (**b**) bacterial attachment rate of the coating.

At the initial stage of marine fouling organism attachment, microscopic fouling organisms such as marine bacteria accumulate adhesion on the surface of the object and grow fixed on the surface of the object by secreting extracellular polymer (EPS), forming a biofilm in a short period of time, which provides the conditions for the attachment growth of large fouling organisms [5]. We evaluated the anti-bacterial adhesion performance of the coating by marine bacterial biofilm adhesion experiments, and the results are shown in Figure 8. Figure 8a shows the original image of bacterial colonies, which visually shows that a large number of colonies were attached to the PDMS coating. The colony attachment rate was obtained by calculation, as shown in Figure 8b. The results show that the addition of SLAP, g-C_3_N_4_, and SLAP@CN can effectively inhibit the growth of bacterial adhesion and enhance the anti-bacterial adhesion performance of PDMS. Among them, the anti-bacterial adhesion performance of SLAP was better than that of g-C_3_N_4_, but the anti-bacterial adhesion performance of these two powders was worse than that of SLAP@CN. This is because SLAP@CN possesses the fluorescent sterilization performance of SLAP and the photocatalytic sterilization performance of g-C_3_N_4_. In addition, the fluorescence emitted by SLAP in SLAP@CN when there is no external light provides a light source for g-C_3_N_4_ to continue its photocatalytic activity for a period of time. Since the ratio of g-C_3_N_4_ and SLAP in SLAP@CN influenced the luminescence intensity and photocatalytic performance of the composite powder, the four SLAP@CN/PDMS differed in their anti-bacterial adhesion performance. Among the SLAP@CN/PDMS composite coatings, the SLAP@CN-1/PDMS bacterial adhesion rate was the largest. Analyzed from the perspective of the surface properties of the coatings, the WCA and SE of SLAP@CN-1/PDMS were very close to the other three composite coatings, and the roughness was also smaller. Therefore, the anti-bacterial adhesion performance of SLAP@CN-1/PDMS was mainly related to the ratio of the composite powder. The large bacterial adhesion rate is due to the lowest content of g-C_3_N_4_ in SLAP@CN-1/PDMS, which has a worse photocatalytic anti-bacterial performance than the remaining three coatings, and the coating relies more on fluorescence to sterilize in a dark environment. At the same time, g-C_3_N_4_ coating on the surface of SLAP blocked part of the fluorescence, resulting in a decrease in fluorescent antifouling performance. g-C_3_N_4_ was the best anti-bacterial adhesion performance in SLAP@CN-2.5/PDMS, indicating that this ratio of the composite powder could more fully utilize the fluorescence and photocatalytic sterilization performance.

### 3.7. Anti-Bacterial Mechanism of Coatings

**Figure 9:**
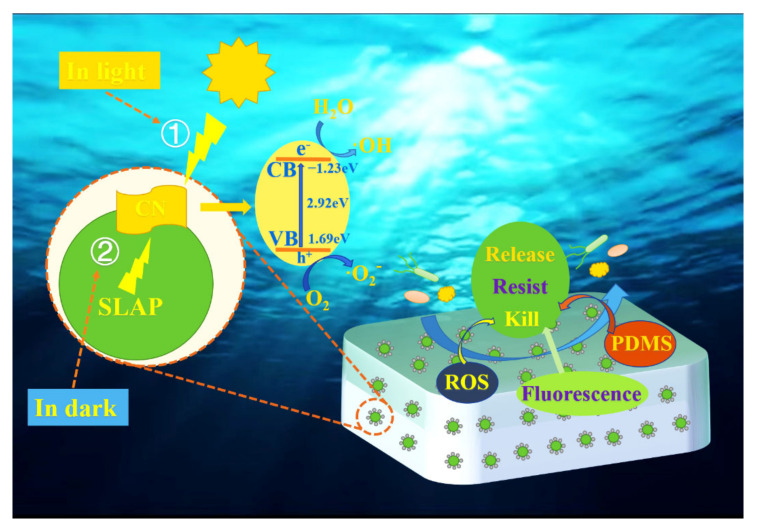
Schematic diagram of the attachment mechanism of anti-marine bacteria.

The anti-marine bacterial adhesion mechanism of the coating mainly combines fluorescence sterilization, photocatalytic sterilization, fluorescence-driven photocatalytic sterilization, and the low surface energy anti-bacterial of silicone, as shown in Figure 9. The bacterial adhesion rate of SLAP@CN/PDMS is lower than that of SLAP/PDMS and g-C_3_N_4_/PDMS. On the one hand, it is due to the combination of fluorescence and photocatalytic anti-bacterial properties of SLAP@CN/PDMS. On the other hand, it is because SLAP provides a light source for g-C_3_N_4_ in a dark environment, which gives it fluorescence-driven photocatalytic properties. The analysis of the fluorescence anti-bacterial mechanism shows that the bacterial adhesion rate of SLAP/PDMS is lower than that of PDMS, which proves that the fluorescent coating has good antifouling properties. The blue light in visible light has a sterilizing effect, and the biofilm and protein formation of marine bacteria are inhibited under the action of blue light with a wavelength of 405–470 nm. Moreover, such blue light also inactivates Gram-negative bacteria and also inhibits the growth of *E. coli* T2 phage and eventually inactivates them [51,52]. Therefore, SLAP emits light sources with wavelengths of 420–530 nm that are sterilizing and anti-bacterial. According to relevant literature reports, the greater the luminescence intensity of the coating is, the better the anti-bacterial and anti-microbial adhesion properties of the coating become [53]. It is worth noting that the content of g-C_3_N_4_ in the coating affects the luminescence performance of the coating. The greater the content of g-C_3_N_4_ in SLAP@CN is, the more g-C_3_N_4_ obscures the light emitted by SLAP, leading to a decrease in the luminescence intensity of the coating, which reduces the fluorescent anti-bacterial performance.

Mechanistic analysis of photocatalytic sterilization: The degradation performance of the composite powder against pollutants under visible light irradiation was verified by RhB degradation experiments. Photocatalytic performance in SLAP@CN was provided by g-C_3_N_4_. g-C_3_N_4_ has a thinner structure after liquid-phase exfoliation, which shortens the distance and time for photogenerated carriers to migrate to the surface. At the same time, the thinner lamellar structure exposes more active sites for more efficient utilization of visible light [54]. Although the band gap energy of the exfoliated g-C_3_N_4_ increases, the exfoliated g-C_3_N_4_ still has higher visible light photocatalytic activity according to previous studies [42]. In addition, there is an interfacial interaction between SLAP@CN that reduces the complexation rate of photogenerated carriers. The combined effect of these factors allows more photogenerated carriers to migrate to the catalyst surface for redox reactions and enhances photocatalytic sterilization performance. g-C_3_N_4_ generates photogenerated carriers that migrate to the catalyst surface and undergo redox reactions with H_2_O, H^+^, and OH^−^ adsorbed on the surface, producing ROS with redox properties (·OH, HO_2_^−^, and O_2_^−^, etc.). ROS not only reduce the vital activity of bacteria but also cause damage to their cell membrane and cell wall structure, leading to their inactivation [55]. In addition, ROS produced by photocatalytic reactions causes oxidative damage to biological macromolecules (proteins, lipids, DNA and RNA, etc.) and eventually kills bacteria [56]. Therefore, the marine bacterial adhesion experiment was performed at a light intensity of 2150 lux for 12 h. g-C_3_N_4_ has good photocatalytic activity and effectively kills the bacteria attached to the coating surface. The bacterial adhesion rate of g-C_3_N_4_/PDMS in the results of the marine bacterial adhesion experiment was less than that of PDMS, which proved that g-C_3_N_4_ in the composite powder could effectively inhibit the adhesion of marine bacteria.

Analysis of the performance of fluorescence-driven photocatalytic sterilization: Photocatalytic sterilization plays a major role when the external light source is sufficient and a secondary role when fluorescence is the light source, which reflects the synergy between photocatalytic sterilization and fluorescence sterilization. The essence of fluorescence-driven photocatalytic sterilization is also g-C_3_N_4_ photocatalytic sterilization. The analysis of PL and UV–Vis results shows that the fluorescence emission wavelength can match the g-C_3_N_4_ light absorption wavelength, which is necessary to excite g-C_3_N_4_ to produce photocatalytic activity. The UV–Vis results showed that the compounding of g-C_3_N_4_ with SLAP could enhance the visible light absorption ability of SLAP. The enhanced visible light absorption ability is beneficial for SLAP to store energy under light conditions, which enables it to perform better afterglow performance in a dark environment. The RhB experiment shows that the composite powder exhibits continuous degradation ability to RhB after the light source is turned off. The degradation rate decreases with time, and this result coincides with the afterglow decay curve of SLAP. According to the reference literature [57], it is known that the photocatalytic reaction rate is related to the absorption and utilization of the light by photocatalytic materials, and increasing the light intensity can effectively increase the photocatalytic degradation reaction rate. When the light intensity is low, the photocatalytic reaction rate is proportional to the light flux. When the light intensity is higher than one solar energy equivalent, the photocatalytic reaction rate increases with the square root of the luminous flux. Therefore, it can be known that the fluorescence intensity is proportional to the photocatalytic rate. From the afterglow curve, it can be seen that the SLAP afterglow intensity showed an exponential decrease after stopping the light. The afterglow intensity was greater in the first 20 min, so the degradation rate was greater. After 20 min, the afterglow intensity decayed slowly, so the fluorescence-driven g-C_3_N_4_ photocatalytic activity decreased. However, the rate of RhB degradation by the composite powder in the dark environment was still 10.1 times higher than that of g-C_3_N_4_. Therefore, the coating was able to continue to provide the light source for g-C_3_N_4_ in the dark environment, extending the photocatalytic sterilization performance for a period of time.

Analysis of the antifouling properties of silicones: Silicone antifouling mainly relies on its own low SE and smooth surface properties [58]. The experimental results showed that the SE of the coating did not change significantly after the composite powder was added to PDMS, and the SE of the coating was between 18.6 and 18.9 mJ/m^2^. Since seawater with high SE cannot wet the surface with low SE [33], some microorganisms in seawater could not contact and attach to the surface of the coating, thus enhancing the anti-bacterial adhesion performance of the coating. In conclusion, the anti-bacterial adhesion performance of the coating is mainly related to the nature of the silicone coating, the blue light emitted by SLAP, and the photocatalytic sterilization of g-C_3_N_4_. The anti-bacterial adhesion performance of the coatings was related to the amount of g-C_3_N_4_ in the SLAP@CN composite powder. Too much g-C_3_N_4_ reduced the fluorescent anti-bacterial performance, while too little g-C_3_N_4_ affected the photocatalytic sterilization performance. The relative adhesion rate of SLAP@CN-2.5/PDMS was the smallest among all coatings. It was shown that the coating had the best overall antifouling performance when g-C_3_N_4_ with a mass fraction of 2.5% was covered on the SLAP surface. The three antifouling mechanisms, fluorescent antifouling, photocatalytic antifouling, and fluorescence-driven photocatalysis, played an important role in enhancing the static antifouling performance of the coating in synergy with each other. The catalytic antifouling effect is more obvious when the external light is strong, while fluorescent antifouling and fluorescence-driven photocatalytic antifouling play an important role simultaneously in the dark environment.

## 4. Conclusions

In conclusion, based on fluorescence antifouling and photocatalytic antifouling, SLAP@CN/PDMS composite antifouling coating was designed and prepared in this paper. The antifouling coating introduced fluorescent antifouling, photocatalytic antifouling, and fluorescence-driven photocatalytic antifouling mechanisms based on the low surface energy antifouling mechanism of organosilicon to achieve “all-weather” photocatalytic antifouling. The results show that g-C_3_N_4_ is successfully coated on the SLAP surface and forms a heterogeneous interface. The visible light absorption range decreases with the increase of g-C_3_N_4_ content in SLAP@CN, while E_g_ increases on the contrary. The luminescence intensity of the coatings decreases with increasing g-C_3_N_4_ content in SLAP@CN/PDMS. The composite coatings all have low surface energy and roughness. The photocatalytic performance of the composite powders was analyzed experimentally, and the blue light emitted from SLAP when the light disappeared was able to drive the photocatalytic reaction of g-C_3_N_4_ and enhance the photocatalytic performance. The coating showed good anti-marine bacteria adhesion performance, and the coating was able to effectively inhibit the adhesion of marine bacteria on the coating surface, where the anti-bacteria adhesion performance of SLAP@CN-2.5/PDMS was improved by nearly 19 times. Further analysis of the results shows that photocatalytic sterilization played a more important role when the external light source was sufficient, and g-C_3_N_4_ had strong photocatalytic activity to enhance the anti-bacterial performance of the organosilicon coating. When there is no external light source, fluorescence sterilization plays a more important role. Blue light emitted by SLAP can inactivate some bacteria, and it can also provide the light source for g-C_3_N_4_ in the dark environment so that it can maintain its photocatalytic activity and effectively improve the anti-bacterial performance of the silicone coating. The composite coating itself has low surface energy, which makes it more difficult for fouling organisms to adhere to the coating surface. Therefore, the introduction of SLAP@CN into the silicone antifouling coating can effectively enhance antimicrobial performance. The development of this “green” and “clean” composite antifouling coating provides a reference for marine environmental protection antifouling coatings.

## Data Availability

Not applicable.
